# Emerging Evidence for cAMP-calcium Cross Talk in Heart Atrial Nanodomains Where IP_3_-Evoked Calcium Release Stimulates Adenylyl Cyclases

**DOI:** 10.1177/25152564211008341

**Published:** 2021-04-25

**Authors:** Rebecca-Ann B. Burton, Derek A. Terrar

**Affiliations:** Department of Pharmacology, 6396 University of Oxford, Oxford, UK

**Keywords:** IP_3_, IP_3_R, adenylyl cyclase, atria, calcium, nanodomains, cAMP

## Abstract

Calcium handling is vital to normal physiological function in the heart. Human atrial arrhythmias, eg. atrial fibrillation, are a major morbidity and mortality burden, yet major gaps remain in our understanding of how calcium signaling pathways function and interact. Inositol trisphosphate (IP_3_) is a calcium-mobilizing second messenger and its agonist-induced effects have been observed in many tissue types. In the atria IP_3_ receptors (IR_3_Rs) residing on junctional sarcoplasmic reticulum augment cellular calcium transients and, when over-stimulated, lead to arrhythmogenesis. Recent studies have demonstrated that the predominant pathway for IP_3_ actions in atrial myocytes depends on stimulation of calcium-dependent forms of adenylyl cyclase (AC8 and AC1) by IP_3_-evoked calcium release from the sarcoplasmic reticulum. AC8 shows co-localisation with IP_3_Rs and AC1 appears to be nearby. These observations support crosstalk between calcium and cAMP pathways in nanodomains in atria. Similar mechanisms also appear to operate in the pacemaker region of the sinoatrial node. Here we discuss these significant advances in our understanding of atrial physiology and pathology, together with implications for the identification of potential novel targets and modulators for the treatment of atrial arrhythmias.

## Introduction

Calcium signaling is a key contributor to the normal physiological functioning of the heart. Amongst the many roles calcium plays, its function as the essential ion for excitation-contraction coupling (ECC), an intracellular process that links cardiomyocyte depolarisation to contraction is of prime importance. In the heart this process depends on calcium entry via calcium channels in the sarcolemmal membrane leading to release of additional calcium from the sarcoplasmic reticulum (SR) in a process described as calcium-induced-calcium-release (CICR).

Abnormalities of the various components affecting calcium handling which facilitate and control ECC are widely recognized as significant contributors to the contractile dysfunction of the failing heart. In addition, defects in calcium handling are increasingly reported as mediators of certain forms of abnormal cardiac electrical rhythms (Scoote and Williams, 2004; Nattel et al., 2008; Blum et al., 2009). Abnormal rises in intracellular calcium (calcium) concentrations can be highly localised in different spatial domains with dimensions ranging from micron to submicron, or these changes in calcium concentration can be propagated as intra- and intercellular waves spreading over much greater distances (Allbritton and Meyer, 1993).

In addition to the essential actions of calcium in CICR, calcium plays a central role in cell signaling including regulation by calcium/calmodulin-dependent protein kinase II (CaMKII), inositol-1,4,5-trisphosphat (IP_3_), cyclic ADP ribose, and nicotinic acid adenine dinucleotide phosphate (NAADP) (Berridge et al., 2003; Terrar, 2020), and phosphorylation/dephosphorylation events occur at a plethora of proteins involved in each pathway.

The focus of this review is on localised interactions between IP_3_ and cAMP signaling pathways, particularly in the heart. In discussing evidence in a variety of tissues, it has previously been argued that rises in cAMP concentration may be spatially confined because components of the cAMP signaling pathway are anchored and highly localised in specific subcellular regions (recently reviewed in Zaccolo et al., 2021). Study of these highly localised changes in cAMP has been facilitated by the use of genetically encoded probes to give a signal dependent on fluorescence energy transfer (FRET), and this occurs over a nanometer scale (Adams et al., 1991; Zaccolo and Pozzan, 2002). Studies in neonatal ventricular myocytes have shown that highly localised changes in cAMP concentration can be detected by FRET probes targeted to specific multiprotein complexes that are less than 300 nm apart (Surdo et al., 2017). In retinal axon arbors it has been found that cAMP changes induced by ephrin-A5 occur beneath the surface membrane in regions of membrane where lipid rafts occur, but not in non-raft regions, and this has been taken to provide further evidence of cAMP signaling on a nanometer scale since the dimensions of lipid rafts are in the range 20-200 nm (Averaimo et al., 2016; Zaccolo et al., 2021). Highly localised domains centred on adenylyl cyclases (AC) which catalyse the synthesis of cAMP have also been reviewed in Cooper and Tabbasum (2014). An important aspect of this localised cAMP signaling is the attachment of different isoforms of PKA to specific anchoring proteins, and such macromolecular complexes may also include phosphodiesterases and phosphatases (Dessauer, 2009; Bers et al., 2019). In view of this extensive evidence it seems reasonable to refer to the loci of these cAMP signaling mechanisms as ‘nanodomains’ to signify regions that are substantially smaller than the dimensions of the cardiac sarcomere contractile unit (slightly less than 2 μm under resting conditions). The signaling mechanisms that depend on IP_3_ are also likely to include highly localised domains depending on the spatial organization of IP_3_ receptors (IP_3_Rs), as discussed in more detail below.

## IP3Rs in the atria

There exist many differences between atrial and ventricular cardiomyocytes in terms of the ECC process. These differences have been recently highlighted in the atria in terms of the handling of calcium ions by different intracellular compartments (Blatter, 2017; Capel et al., 2021). In 1983, (Streb et al., 1983) demonstrated release of calcium from a non-mitochondrial intracellular store by IP_3_ in pancreatic acinar cells. IP_3_ has emerged as a ubiquitous intracellular messenger, releasing calcium from intracellular stores through the activation of inositol trisphosphate receptors (IP_3_Rs) (Kockskämper et al., 2008). One of the important actions of IP_3_ in the atria is to increase the force of contraction. Atrial myocytes possess an abundance of IP_3_Rs when compared with ventricular myocytes (Lipp et al., 2000; Kockskämper et al., 2008). In rat and rabbit myocytes IP_3_R expression is ∼3.5-10 times larger in atrial myocytes compared to ventricular myocytes (Lipp et al., 2000; Domeier et al., 2008). IP_3_Rs in atrial myocytes appear to be much more important for the acute regulation of the calcium transients that control contraction (Lipp et al., 2000) than is the case for ventricular myocytes. In addition, these IP_3_Rs seem to be highly localised in atrial myocytes in the junctional SR just beneath the surface membrane rather than in the non-junctional SR (Lipp et al., 2000), consistent with the possibility of IP_3_ signaling in nanodomains. Although there has been a substantial increase in our understanding of these pathways in the last 15 years, the emphasis has been on interactions between IP_3_ dependent calcium release and ryanodine receptor (RyR) dependent calcium release (Nosek et al., 1986; Kentish et al., 1990; Fabiato, 1992; Kockskämper et al., 2008). In terms of IP_3_Rs and pathology, studies more recently have focused on the IP_3_ receptor-induced release (IICR) in heart failure (HF) in rabbit atrial cells. These observations showed that in HF atrial myocytes there was an increase in diastolic [calcium]_i_ and that action potential (AP)-induced calcium transients (CaTs) were larger in amplitude. The larger CaTs were due to IICR and reduced mitochondrial calcium buffering. These observations are consistent with a reduced mitochondrial density as well as calcium uptake capacity in HF. Interestingly, the central regions of the non-junctional SR displayed more frequent calcium puffs in the HF compared to normal cells (Hohendanner et al., 2015).

## cAMP – calcium cross-talk in nanodomains

As mentioned above, adenylyl cyclases (AC) are enzymes that play key regulatory roles catalysing the production of cAMP (Sassone-Corsi, 2012). There are nine isoforms of AC that are membrane bound (Dessauer et al., 2017), and an additional soluble enzyme (AC10) is regulated by the concentration of bicarbonate anions (Wiggins et al., 2018). Two AC isoforms, AC5 and AC6 have been shown to be inhibited by calcium, while AC1 and AC8 are stimulated by calcium (Cooper and Tabbasum, 2014; Zaccolo et al., 2021). AC3 shows complex regulation by calcium since it can be stimulated via calcium/calmodulin (though with a substantially lower calcium-sensitivity than AC1 and AC8, see Cooper et al. (1995)), but is inhibited by CaMKII (Wei et al., 1998). This enzyme seems to be a key component of olfactory mechanisms where the calcium-dependent inhibition may be important for termination of the response (Wong et al., 2000). Other AC isoforms seem to show little or no sensitivity to calcium. In pituitary-derived GH_3_B_6_ cells stimulation by thryrotropin releasing hormone was shown to give rise to localised changes in cAMP concentration (Wachten et al., 2010). It was suggested that calcium release via IP_3_Rs in these pituitary-derived cells might inhibit AC5 and 6, and also stimulate AC8, with these enzymes being located in different regions of the surface membrane (Wachten et al., 2010).

**Figure 1.**
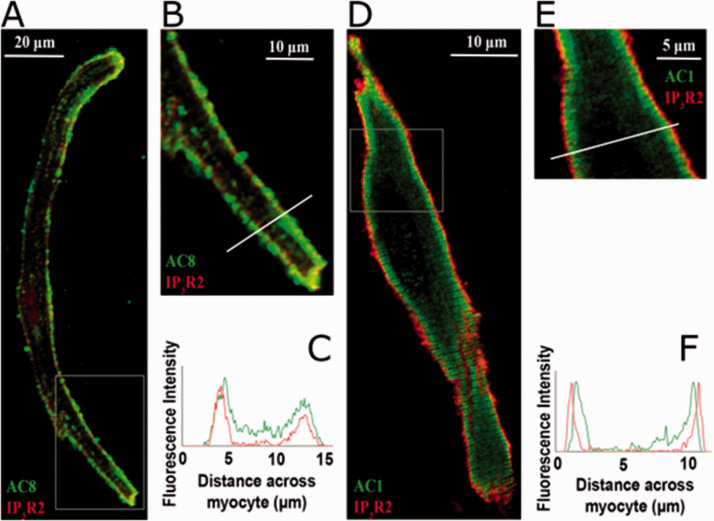
Co-localisation of IP_3_ receptors with calcium-stimulated adenyl cyclases identified by immunocytochemistry. A: An atrial myocyte in which IP_3_R2s are labelled red while AC8 is labelled green. B: The cellular location of these two proteins at higher magnification. C: A plot of intensity of red and green fluorescence against distance across the cell at the line at right angles to the long axis of the cell shown in panel B. D: An atrial myocyte in which IP_3_R2s are again labelled red while AC1 is labelled green. E: The cellular location of IP_3_R2 and AC1 at higher magnification, while F shows a plot of intensity of red and green fluorescence against distance across the cell at the line at right angles to the long axis of the cell shown in panel E. Note that there was colocalisation of IP_3_R2 and AC8, while AC1 appeared to be located on the cytosolic side of junctional SR. From Capel et al. (2021).

**Figure 2.**
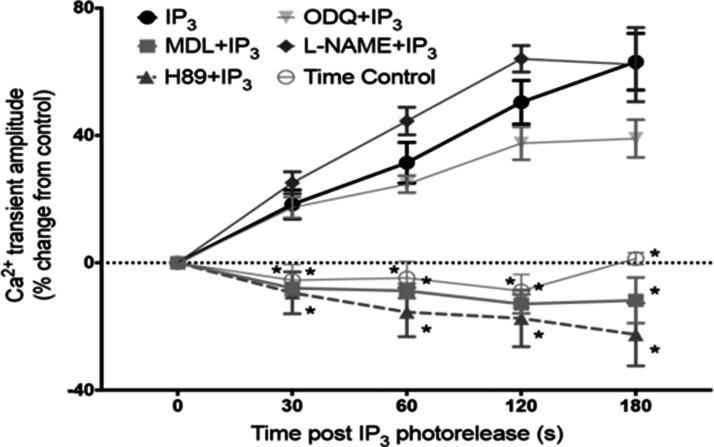
Effects of IP_3_-evoked calcium release on calcium transients (CaT) depend on adenylyl cyclases and PKA. A plot of changes in amplitude of calcium transients (CaT) accompanying action potentials (stimulation frequency, 1 Hz) measured as fluo-5 fluorescence following intracellular photorelease of IP_3_ from a caged compound. In the absence of drugs photorelease of IP_3_ led to a progressive increase in CaT amplitude over the 3 min time period shown. These effects of photoreleased IP_3_ were suppressed when adenylyl cyclases were inhibited by MDL 12-330A (3μmol/L), and when PKA was inhibited by H89 (1μmol/L). The time control for changes in CaT amplitude in the absence of photorelease of IP_3_ showed a small progressive decline. The increase in CaT amplitude following photorelease of IP_3_ seemed little if at all affected by inhibition of eNOS by L-NAME or by inhibition of guanylyl cyclase by ODQ, showing that NO signaling seemed not to be a major contributor to the changes in CaT amplitude that resulted from IP_3_-evoked calcium release from the SR. From Capel et al. (2021).

**Figure 3.**
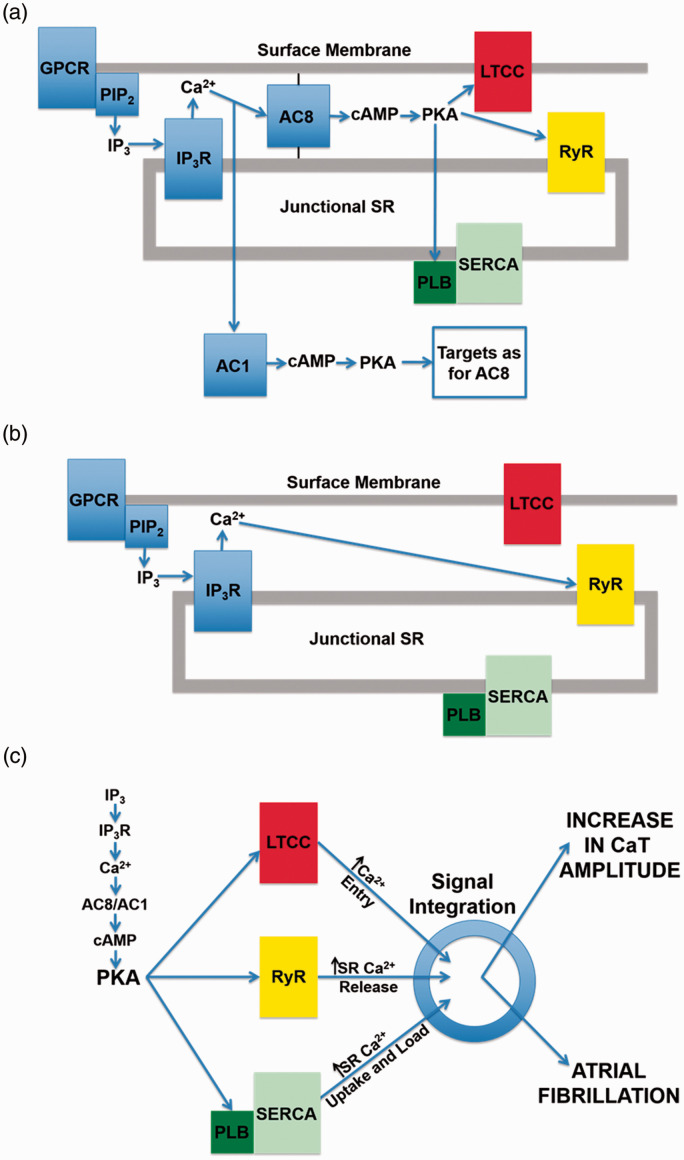
Schemes showing possible mechanisms of action of IP_3_-evoked calcium release (a) Scheme showing location of IP_3_ receptors (IP_3_R) in junctional SR, together with proteins relevant to the newly proposed signaling mechanism. IP_3_-evoked calcium release from SR stimulates calcium-activated adenylyl cyclases (AC8 and AC1) leading to production of cAMP and PKA, which in turn phosphorylates target proteins, L-type calcium channels (LTCC), ryanodine receptors (RyR) and phospholamban (PLB) associated with sarcoplasmic-endoplasmic reticulum calcium ATP-ase (SERCA). IP_3_ is produced in response to stimulation of a G-protein coupled receptor (GPCR) leading to splitting of PIP2 into diacylglycerol and IP_3_. AC8 is shown anchored to the surface membrane and to junctional SR since current immunocytochemistry with light microscopy does not allow us to distinguish between a location in surface membrane caveolae or SR. This designation is intended to emphasise that AC8 shows colocalisation with IP_3_Rs (as shown in Figure 1), so that if AC8 were in caveolae then presumably this component of the surface membrane must be in very close proximity to the IP_3_Rs in the junctional SR. AC1 appears to be anchored at an intracellular site but the precise location of this attachment is currently unclear. (b) Previously proposed simple scheme showing effect of calcium release from IP_3_Rs to enhance calcium release through neighbouring RyRs without involvement of calcium-activated adenylyl cyclases. Note that in the experiments of Capel et al. (2021) shown in Figure 2 there was little or no effect of photoreleased IP_3_ on the amplitude of CaTs when adenylyl cyclase or PKA were inhibited. (c): Scheme following on from Figure 3(a) showing functional consequences of production of PKA with subsequent phosphorylation of the same target proteins. It is again proposed that IP_3_-evoked calcium release activates AC8 and AC1 to produce cAMP and therefore activation of PKA. Phosphorylation of the target proteins by PKA leads to additional calcium entry via LTCC, additional calcium release from the SR via RyR, together with an increased rate of calcium uptake and an increased SR calcium load resulting from enhanced SERCA activity after phosphorylation of PLB. Cellular processes integrate these changes in calcium fluxes first to produce an increase in the amplitude of the calcium transient accompanying action potentials (CaT), and then to cause atrial fibrillation when IP_3_ and PKA levels are excessively high.

In the heart, AC5 and AC6 have been recognized as the predominant isoforms in ventricular cardiomyocytes (Guellich et al., 2014), and calcium-dependent inhibition of cAMP production by these enzymes can exert a regulatory influence on L-type calcium channels (Yu et al., 1993). However, in atrial and sino-atrial node cells there is a functionally important expression of the calcium-stimulated isoforms AC1 and AC8 (Mattick et al., 2007; Younes et al., 2008; Collins and Terrar, 2012). We are not aware of any previous work proposing a link between cAMP and IP_3_ signaling mechanisms in cardiac myocytes. The AC8 isoform appears to show co-localisation with IP_3_R in immunocytochemistry experiments, although the limits of conventional light microscopy do not allow us to determine the exact position of AC8, which might be in caveolae in the surface membrane overlapping with junctional SR, or perhaps in the junctional SR itself (Capel et al., 2021). Whether or not AC8 is located in the surface membrane, it is clear that the membrane geometry ensures that this enzyme subtype is very close to IP_3_Rs. Interestingly, AC1 seems to be located on the intracellular side of the junctional SR (Capel et al., 2021). Key observations from this work are reproduced in Figure 1(A) to (F). Atrial muscle has an ongoing activity of ACs, even in the absence of adrenoceptor stimulation (Vinogradova et al., 2006), while these enzymes are essentially quiescent in the ventricle until adrenoceptors are activated, for example by adrenaline or nerve-released noradrenaline. It is thought that AC1 and AC8 contribute to this ‘resting’ adenylyl cyclase activity in atrial muscle. The central role of calcium ions in the initiation and maintenance of atrial arrhythmias has received much prominence (e.g. (Nattel and Dobrev, 2012; Heijman et al., 2014). Recent observations (Capel et al., 2021), are consistent with a functional interaction between IP_3_ and cAMP signaling that involves calcium-stimulated ACs in the atria and sino-atrial node. These data show downstream effects of IP_3_ photorelease, leading to functional and physiological consequences of IP_3_R opening in cardiac atrial myocytes. The effects of calcium release from IP_3_Rs seem to require modulation of other signaling pathways rather than to be the result of direct effects of this released calcium on RyRs. Key observations shown in Figure 2 support the proposal that IP_3_-evoked calcium release leads to an increase in the amplitude of calcium transients (CaT) accompanying action potentials by stimulation of calcium-activated ACs. Figure 3(a) shows a scheme to account for these observations in which stimulation of AC8 and AC1 by IP_3_-evoked calcium release leads to production of cAMP followed by activation of protein kinase A (PKA) and subsequent phosphorylation of target proteins in the surface membrane (L-type calcium channels) and in the SR (ryanodine receptors, RyR, and phospholamban (PLB) associated with sarcoplasmic-endoplasmic reticulum ATP-ase (SERCA)). Figure 3(b) shows the previously proposed simple scheme in which IP_3_-induced calcium release from IP_3_Rs exerts direct effects on neighbouring RyRs to enhance SR calcium release via this protein. The observations of Capel et al. (2021) reproduced in Figure 2 also show that there were negligible effects of photorelease of IP_3_ on CaT either when adenylyl cyclase was inhibited by MDL-12,330A or when PKA was inhibited by H89, and it therefore appears that any residual direct effects of IP_3_-evoked calcium release on RyRs (as in the scheme in Figure 3(b)) were small under the conditions of the experiments. Another more complex pathway proposed by (Wang et al., 2005) is that IP_3_-evoked calcium release activates endothelial nitric oxide synthase (eNOS) to produce NO followed by activation of guanylyl cyclase to produce cGMP, which in turn reduces phosphodiesterase activity leading to an increase in cAMP. These observations were in cat atrial myocytes, while the observations in guinea pig atrial myocytes (Capel et al., 2021) shown in Figure 2 were not consistent with this scheme since the effects of IP_3_ photorelease were not reduced by inhibitors of eNOS or guanylyl cyclase. It remains to be determined whether species differences alone can account for this apparent discrepancy. The scheme in Figure 3(c) complements Figure 3(a) in showing the functional consequences of phosphorylation by PKA of the target proteins shown in panel (a). Phosphorylation of all three proteins is expected to increase the amplitude of CaTs since calcium entry through LTCCs will be enhanced, calcium uptake into the SR by PLB-modulated SERCA will be increased to achieve a higher intraluminal calcium concentration, and SR calcium release by RyRs will be enhanced. The scheme also shows that excessive stimulation of these pathways will lead to atrial fibrillation, and there may perhaps be additional effects on potassium channels (see below).

It was emphasized in the Introduction that the compartmentalization of cAMP/PKA signaling with tight spatio-temporal control of signal propagation allows for specific responses (Terrin et al., 2006; Zaccolo et al., 2021) and this is also the case in cardiac myocytes (Monterisi et al., 2017; Bers et al., 2019) The strategic localisation of IP_3_Rs on the junctional SR membrane in the vicinity of AC8 and AC1 which are positioned close to the surface membrane of atrial and SAN myocytes (as described above and shown in Figure 1(A) to (F)) favours participation of these enzymes in nanodomain calcium signaling (as shown in the scheme in Figure 3(a)).

## IP_3_ and ACs in atrial arrhythmias

Zima and Blatter (2004) have proposed that IP_3_-evoked release from the SR might ‘facilitate’ CICR by increasing the local calcium concentration close to RyRs (Zima and Blatter, 2004) and this may result in triggering atrial arrhythmias (observed by the generation of spontaneous calcium waves). In cat and rabbit atrial myocytes, endothelin-1 induced calcium alternans may degenerate into arrhythmogenic calcium waves (Pieske and Kockskämper, 2002; Zima and Blatter, 2004). In the human atrial myocardium, endothelin-1 has been shown to induce extra contractions mediated by activation of PLC and IP_3_Rs (Burrell et al., 2000), whilst in isolated human atrial myocytes angiotensin II increased the frequency of spontaneous calcium sparks without modifying SR calcium load (Gassanov et al., 2006). The most common atrial arrhythmia associated with aging is atrial fibrillation (AF). The expression of IP_3_Rs has been shown to increase with age (Kaplan et al., 2007) and their expression is also augmented in dog and human AF (Yamda et al., 2001; Cao et al., 2002; Zhao et al., 2007).

Excessive stimulation of IP_3_Rs can cause atrial arrhythmias (Yamda et al., 2001). This is also the case for excessive stimulation of ACs as shown by (Zhao et al., 2015). Future work on calcium-stimulated ACs has the potential to provide important insights regarding acute mechanisms relating to the initiation of atrial arrhythmias. In addition, intracellular phoshodiesterases are important regulators of cAMP concentrations through degradation of cAMP, reducing protein kinase A (PKA) activity. PDEs have a significant impact on atrial contractile and electrical activity, and may play a role in atrial arrhythmias and affect risk of stroke (e.g. (Molina et al., 2012; Van Wagoner and Lindsay, 2012). Putting these observations in the context of the observations of Capel et al (2021) discussed above showing that actions of IP_3_-evoked calcium release in atria depend on stimulation of the calcium-activated adenylyl cyclases, AC8 and AC1, it seems likely that these enzymes may play a crucial role in mechanisms underlying atrial fibrillation when these pathways are excessively stimulated (see scheme in Figure 3(c)). In addition to effects on the three proteins shown in this scheme, there may perhaps be additional effects mediated by AC8 and AC1 on potassium channels, for example to enhance the amplitude and kinetics of I_Ks_ which is also targeted by PKA (Marx et al., 2002), and the consequent more rapid repolarization might be especially important in atrial fibrillation.

This interpretation requiring a major contribution of AC1 and AC8 to IP_3_-signaling in the atria opens the way for a better understanding of this very important pathology and for development of new treatments and drugs.

## IP_3_ and AC in pacemaking

Before considering possible effects of IP_3_ on pacemaker activity in the sino-atrial node (SAN) it is necessary to give a brief outline of possible timing mechanisms.

**Figure 4.**
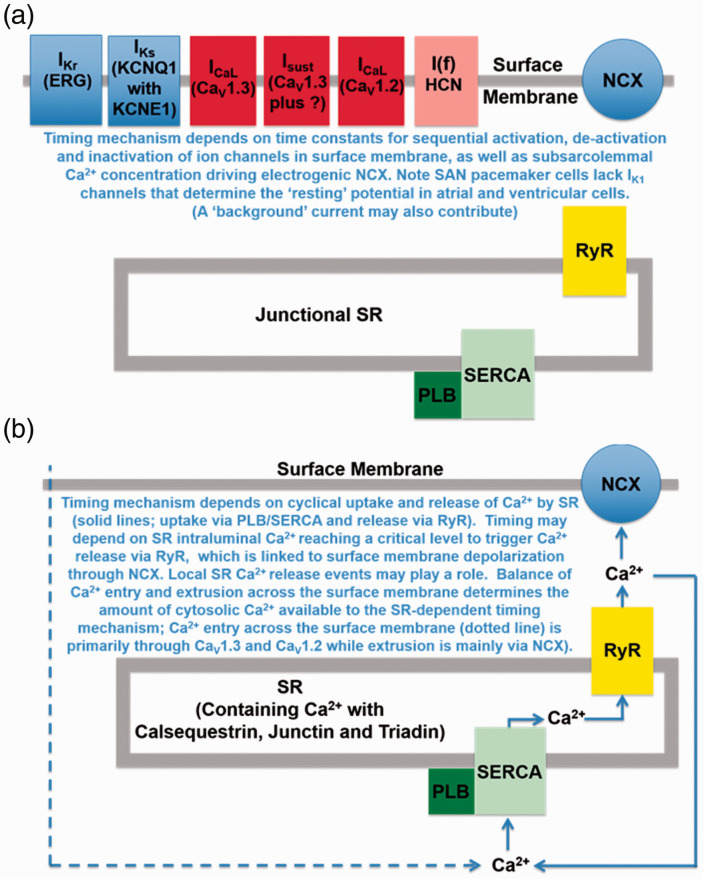
SAN timing mechanisms. (a): Timing mechanism dependent on ionic currents in the surface membrane (‘Membrane Clock’). Activation of potassium channels by the SAN action potential leads to repolarization, but once the most negative potential is achieved de-activation removes their hyperpolarizing influence thus contributing to slow depolarization. Potassium channel currents include I_Kr_ (through ERG channel protein) and I_Ks_ (carried by KCNQ1 with KCNE1, also known as K_V_LQT1 and minK). HCN channels and sustained inward current (through Cav1.3 plus another unknown protein) further contribute depolarizing influences to the slow depolarization. Activation of calcium current through Ca_V_1.3 channels also adds to the later stages of the slow depolarization, which then triggers the action potential upstroke via activation of a combination of Ca_V_1.3 and Ca_V_1.2. The cycle then repeats itself. Note that SAN cells, unlike atrial myocytes, lack the stabilising influence of I_K1_. Electrogenic calcium extrusion via NCX can also contribute to the depolarization, with the amplitude driven by the magnitude of changes in sub-sarcolemmal calcium concentration (see text and Capel and Terrar, 2015). (b): Timing mechanism dependent on SR proteins (‘calcium clock’). For a given cytosolic calcium concentration the speed of calcium uptake by the SR is determined by the activity of SERCA which is itself regulated by phospholamban (PLB). calcium release from the SR back into the cytoplasm is through the RyR protein, with a timing that is presumably driven by events in the lumen of the SR, perhaps involving the calcium concentration reaching a critical level to trigger opening of the RyR channel (see text and Capel and Terrar, 2015). The intraluminal calcium binding protein calsequestrin may play a modulatory influence, together with junctin and triadin. Junctional SR is expected to play an important role, but non-junctional SR cannot be excluded in the SR-dependent timing mechanism, and so in this case the intracellular membrane compartment is labeled simply as SR. Electrogenic NCX is expected to couple the cyclic changes in sub-sarcolemmal calcium concentration to depolarization in the surface membrane.

Pacemaker mechanisms remain controversial and seem to involve integration of two timing mechanisms, one in which the timing mechanism depends on the kinetics of opening and closing of ion channels in the surface membrane (the ‘membrane clock’) and another separate timing mechanism dependent on the kinetics of uptake and release of calcium by the SR (the ‘calcium clock’), as reviewed in (Lakatta et al., 2010), and (Capel and Terrar, 2015). Cyclic activity of the membrane clock results from alternating depolarizing and repolarizing influences, with a timing that depends on sequential activation, de-activation and inactivation of a variety of ion channels (see Figure 4(a)). The normal shape of this cyclic activity is a slow depolarization which triggers a rapid upstroke of the SAN action potential, followed by repolarization back to the most negative potential, and then the cycle repeats itself. Potassium channels activated by the rapid depolarization of the action potential provide the repolarizing influences (carrying I_Kr_ currents through ERG proteins, and I_Ks_ through KCNQ1 combined with KCNE1, also known as K_V_LQT1 and minK), while channels which primarily carry Na^+^ or calcium exert the depolarizing influences, including HCN channels that carry I(f). While in atria the contribution of L-type calcium currents is relatively simple (shown by a single LTCC protein in Figure 3(a)) the contribution of related channels in the SAN is more complex. Rapid depolarization in SAN is provided by two varieties of L-type calcium currents (carried by Ca_V_1.2 which is present throughout the heart, and Ca_V_1.3 channels that are more highly expressed in the SAN, (Mangoni et al., 2003; Mangoni et al., 2006). The slow depolarization results from de-activation of potassium channels thus removing their hyperpolarizing influence, accompanied by activation of a combination of channels with a depolarizing influence, including HCN channels carrying Na^+^, sustained inward current primarily carrying Na^+^ through channels that are thought to be made up of Ca_V_1.3 together with another subunit (Toyoda et al., 2017), and Ca_V_1.3 channels which carry calcium but are activated at more negative potentials than Ca_V_1.2 and so contribute to the later stages of the slow depolarization (Mangoni et al., 2003, 2006). HCN channels are stimulated by sub-sarcolemmal cAMP. Electrogenic sodium-calcium exchange (NCX) also contributes ionic current to the membrane clock. NCX is electrogenic since in simple terms each cycle of exchange extrudes one divalent calcium accompanied by entry of three Na^+^, although other modes may also be possible (Kang and Hilgemann, 2004). A rise in sub-sarcolemmal calcium concentration, whether resulting from calcium entry through surface membrane ion channels or from calcium release from the SR, therefore causes depolarization accompanying calcium extrusion via NCX. These membrane clock components are summarized in Figure 4(a). The major components of the ‘calcium clock’ are shown in Figure 4(b). The cyclic activity in this case results from uptake and release of calcium by the SR, and therefore the timing depends on the speed of uptake by SERCA (modulated by PLB) and may depend on the calcium concentration in the lumen of the SR reaching a critical concentration to trigger opening of RyRs giving rise to SR calcium release (as occurs in ventricular myocytes, Chen et al. (2014). At least some of this SR calcium release may be in the form of local calcium release events (Lakatta et al., 2010). NCX appears to play a major role in coupling the membrane and calcium clocks. The most recent work on intact SAN shows synchronized action potentials emerging from a complex mesh of pacemaker cells in which heterogeneous intracellular calcium signals are proposed to play a major role (Bychkov et al., 2020).

For some time, the role played by IP_3_ as a regulator of cardiac pacemaker activity was controversial (Ju et al., 2011; Vinogradova and Lakatta, 2011). Observations from (Ju et al., 2011) first demonstrated that increasing cytosolic IP_3_ following application of a membrane permeant analogue increased pacemaker activity. In addition, (Kapoor et al., 2015), also clearly showed that IP_3_ signaling can influence pacemaking in murine SAN cells. In their study, the role of IP_3_Rs in pacemaking was investigated in an ‘uncoupled’ pacemaking model (involving a ‘calcium clock’ that is dependent on the timing of calcium uptake and release by the SR) in which the uncoupling from surface membrane activity is achieved by the absence of sodium-calcium exchange (NCX) (Kapoor et al., 2015). This is because calcium release from the SR and the consequent rise in sub-sarcolemmal calcium concentration is normally linked to surface membrane depolarization by electrogenic NCX, and therefore failure to express NCX leads to the observed uncoupling. Phenylephrine (PE) increased the frequency of calcium sparks and waves after 3 min exposure in both wild type and NCX knockout SAN cells, and these effects were reversed with 2-APB (which antagonizes the effects of IP_3_) supporting the hypothesis that IP_3_-evoked calcium release participates in the triggering of calcium release from RyRs, potentiating the calcium clock mechanism of pacemaking. An alternative signaling pathway involving IP_3_ has been shown to play a key role in pacemaker activity in embryonic stem cell-derived cardiomyocytes by Kapur and Banach (Kapur and Banach, 2007). Méry *et al*., earlier also demonstrated in their embryonic cardiac stem cell differentiated model that early pacemaker activity is triggered by that is cycling in and out of the SR (Méry et al., 2005).

Following on from the observations in atrial myocytes discussed above, it has been shown that the calcium stimulated adenylyl cyclases, AC8 and AC1, also seem to be important players in mediating the chronotropic effects of IP_3_ in pacemaker cells (Capel et al., 2021). Key observations are that positive chronotropic effects of alpha adrenoceptor stimulation by phenylephrine, which are mediated by diacylglycerol and IP_3_ (Berridge and Irvine, 1984), are markedly suppressed following inhibition of either adenylyl cyclases (by MDL 12-330A, 3 μmol/L) or PKA (by H89, 1 μmol/L). The observations are therefore consistent with the proposal that positive chronotropic effects of IP_3_ depend on stimulation of calcium-activated adenylyl cyclases (AC8 and AC1) by IP_3_-evoked calcium release from the SR.

**Figure 5.**
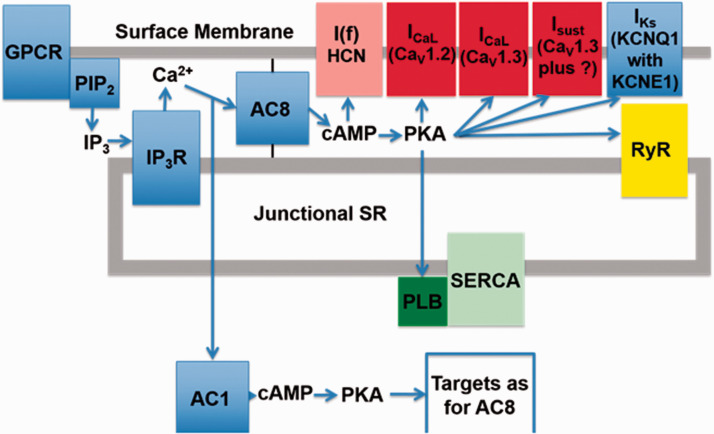
Targets for cAMP and PKA in SAN following activation of AC1 and AC8 by IP3-evoked calcium release. This scheme is analogous to that shown in Figure 3(a) to represent IP_3_ signaling in the atria, but the proposed target proteins in the surface membrane are more diverse including HCN channels directly regulated by cAMP as well as a variety of surface membrane channel proteins phosphorylated by PKA. The targets for PKA again also include the SR proteins, PLB/SERCA and RyR, so that the scheme includes principal components of both the membrane clock and the SR calcium clock shown in Figure 4.

The postulated effects of stimulation of calcium-activated adenylyl cyclases by IP_3_-evoked calcium release are expected to influence components of both the SAN timing mechanisms discussed above (see scheme in Figure 5). Calcium stimulation of AC8 and AC1 is expected to elevate local concentrations of cAMP and to activate PKA. In the case of the membrane clock, I(f) is directly regulated by cAMP, and PKA phosphorylates a variety of membrane proteins. These include L-type calcium channels, but the mechanism is more complex than the model for atria shown in Figure 3 since two types of related protein, Ca_V_1.2 and Ca_V_1.3, are targeted by PKA, with current through Ca_V_1.3 contributing to diastolic depolarization, while currents through both Ca_V_1.3 and Ca_V_1.2 contribute to the upstroke of the action potential. PKA also targets the sustained inward current mechanism (postulated to comprise Ca_V_1.3 together with another protein subunit that changes the ion selectivity from calcium to one that favours Na^+^ (Toyoda et al., 2017)). In addition, potassium channels are influenced by PKA which increases current amplitudes and also speeds channel kinetics (Marx et al., 2002). In the case of the SR calcium clock, PKA phosphorylates both RyR to increase calcium release and PLB/SERCA to enhance calcium uptake. Electrogenic NCX may be important in coupling together the timing mechanisms of membrane and SR calcium clocks, and is also expected to be increased indirectly as a consequence of elevated sub-sarcolemmal calcium following increased calcium entry and release, and perhaps directly by PKA-mediated phosphorylation.

## Additional aspects of nanodomain signaling involving calcium stimulated ACs and other mechanisms in atria and SAN

**Figure 6.**
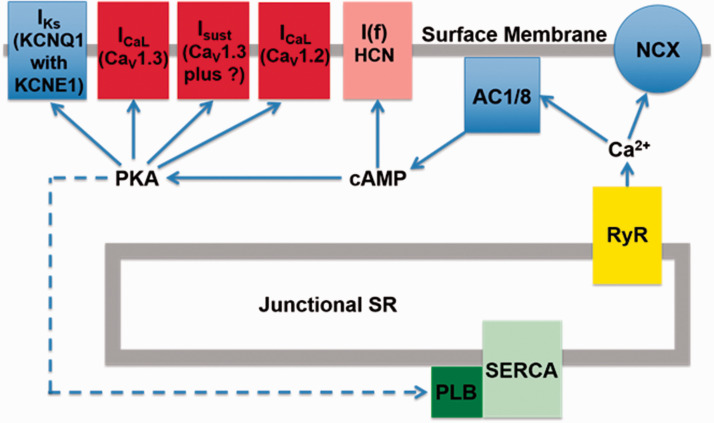
Possible effects of SR calcium release via RyR on surface membrane ionic currents controlling membrane clock in SAN. Scheme building on observed effects of SR calcium release via RyR on L-type calcium currents (Collins and Terrar, 2012) through Ca_V_1.2 and mediated by calcium stimulated adenylyl cyclases (AC8 and AC1). In SAN node, evidence also supports effects on HCN via direct effects of cAMP, and is likely also to involve other PKA targets such as Ca_V_1.3, sustained inward current and potassium channels (see text). For simplicity in this scheme AC8 and AC1 are both shown close to the surface membrane, although it should be noted that the observations in Figure 1 show that AC1 appears to be on the intracellular side of junctional SR, while the location of AC8 appears to overlap with junctional SR.

Although current interpretations of pacemaker activity require an integration of membrane and calcium clocks (Lakatta et al., 2010; Capel and Terrar, 2015), the two mechanisms are often thought of as interacting but distinct. However, it seems likely that calcium release via RyRs may not only play a key role in the SR calcium clock and provide a link to surface membrane depolarization via NCX, but may also exert a major influence on membrane proteins via stimulation of calcium-activated adenylyl cyclases. This is analogous to the proposed effects of IP_3_-evoked calcium release on AC8 and AC1 discussed above, and provides another example of signaling in nanodomains. This aspect of calcium release through RyRs leading to stimulation of AC1 and AC8 seems to apply to both SAN and atria. In the case of regulation of L-type calcium currents in atria, it has been shown that suppressing SR function with ryanodine and thapsigargin reduces calcium current amplitude, and the evidence supports the proposal that SR calcium release stimulates AC1 and AC8 to bring about these effects (Collins and Terrar, 2012). It seems likely that similar mechanisms involving L-type calcium channels also operate in SAN. Evidence supporting this proposal was based on the use of ryanodine, cyclopiazonic acid (an inhibitor of SERCA), selective blockers of I(f), and MDL-12,330A to inhibit adenylyl cyclase, and was consistent with the hypothesis that SR calcium release via RyRs in SAN also influences the amplitude of I(f), at least in part by stimulation of calcium-activated adenylyl cyclases. These effects appeared to be important both in the absence and presence of beta-adrenoceptor stimulation by isoprenaline (Nazarov et al., 2015). While the evidence provided support for an important effect of SR-released calcium on I(f), there were major additional effects of inhibition of SERCA by cyclopiazonic acid even when most or all I(f) currents were blocked, and it was suggested that stimulation of calcium activated adenylyl cyclases by calcium released from the SR contributed to these effects through actions at other potential targets, including PLB/SERCA, L-type calcium channels (Ca_V_1.2 and Ca_V_1.3), sustained inward current and potassium channels (Nazarov et al., 2015). These proposals for SAN are summarized in Figure 6. Such a mechanism could also further amplify the actions of IP_3_-evoked SR calcium release following effects of PKA on neighbouring RyR as in schemes 3(a) and 5. There may be other related aspects of nanodomain signaling to be uncovered in the near future since it has been shown that nicotinic acid dinucleotide phosphate (NAADP) can increase the amplitude of CaTs in atria by an effect that depends on calcium release from acidic stores that are thought to be lysosomes or similar structures (Collins et al., 2011). NAADP provokes calcium release via two pore channels (TPC2) in the membranes of these lysosome-related structures (Capel et al., 2015), which are located close to the SR (Aston et al., 2017). NAADP actions contribute to actions of isoprenaline on beta-adrenoceptors in atria (Collins et al., 2011; Capel et al., 2015). The effects of NAADP-stimulated calcium release from the lysosome-related structures are thought to require stimulation of CaMKII to increase CaT by enhancing calcium uptake into the SR (Capel et al., 2015). Synthesis of NAADP in the heart seems to involve the enzyme CD38 (Lin et al., 2017). Recent preliminary work supports the proposal that a similar NAADP-dependent mechanism contributes to regulation of pacemaker activity in SAN (Capel et al., 2020). This work on the cardiac actions of NAADP therefore provides further support for nanodomain signaling in atrial myocytes and SAN, in this case involving calcium release from lysosome-related structures close to SR and subsequent activation of CaMKII to increase the amount of calcium loaded into the SR.

## Other possible interactions between IP_3_ and cAMP systems

Work in a variety of non-cardiac cells has identified an additional possible mechanism for interactions between cAMP and IP_3_ pathways in which IP_3_Rs are sensitized to stimulation by IP_3_ following phosphorylation by PKA, or by the direct action of high concentrations of cAMP (Taylor, 2017). In these experiments IP_3_Rs continue to be activated by IP_3_ in the absence of cAMP or phosphorylation by PKA (Burgess et al., 1991; Tovey et al., 2008; Betzenhauser et al., 2009; Taylor, 2017). Enhancement of calcium release following phosphorylation of IP_3_R2 seems to be particularly important at low IP_3_ concentrations, while at 1 µM IP_3_ and above the sensitivity of IP_3_R2 seemed to be unaffected by PKA (Betzenhauser et al., 2009). Whether these mechanisms occur in heart cells has yet to be established, and if present might complement or amplify the effects of calcium-activated adenylyl cyclases stimulated by IP_3_-evoked calcium release described above. Emerging evidence proposes that abnormal calcium signals from the IP_3_R1 are closely associated with human brain pathology (Hisatsune and Mikoshiba, 2017). cAMP plays a role in different forms of memory-related, long-term plasticity (Blum et al., 2009). In neurons, activity dependent cAMP synthesis is primarily mediated by membrane bound calcium/calmodulin-stimulated ACs (Vadakkan et al., 2006). Many types of ACs have been shown to be present in the brain (AC 1–9 isoforms; See reviews (Xia and Storm, 1997; Sunahara and Taussig, 2002). AC1 and AC8, are activated by calcium through the calcium-binding protein calmodulin (Xia and Storm, 1997). AC1 contributes to behavioural sensitization in chronic pain animal models (Wei et al., 2002; Vadakkan et al., 2006; Wang et al., 2011) and AC8 is less sensitive to calcium than AC1 (Villacres et al., 1995). Bernabucci and Zhao (2016), demonstrated calcium-stimulated AC8 is required to sustain the long-lasting anxiety caused by repeated elevated plus-maze testing. IP_3_R1 is dominantly expressed in the brain and is important for brain function. It would be interesting to see whether these observed interactions between IP_3_ and calcium-stimulated AC’s occur in brain cells.

It should also be mentioned that there is another pathway with the potential to bring together two different calcium signaling mechanisms, since calcium and calmodulin-dependent protein kinase II (CAMKII) has been recognized as an accessory protein of IP_3_Rs in nuclear membranes of ventricular myocytes (Bare et al., 2005).

## Summary

The above discussion highlights and sets in context the recent observations showing interactions between cAMP and calcium signaling mechanisms in which cross talk between these pathways occurs in heart atrial nanodomains as a consequence of stimulation of calcium-activated adenylyl cyclases (AC8 and AC1) by IP_3_-evoked calcium release from the SR. These interacting signaling pathways lead to increases in the amplitude of CaTs triggered by action potentials, and AC8 and AC1 enzymes stimulated by IP_3_-evoked calcium release are thought to be important newly identified components of mechanisms underlying atrial fibrillation. Similar mechanisms also make a major contribution to chronotropic effects of IP_3_ in pacemaker cells of the SA node. These novel interactions between IP_3_ and adenylyl cyclases in cardiac nanodomains are clearly of fundamental importance to understand the normal physiology of atrial muscle, particularly concerning differences from ventricular muscle. This improved understanding is essential if appropriate novel treatments for atrial diseases are to be found.

## References

[bibr1-25152564211008341] Adams SR Harootunian AT Buechler YJ Taylor SS Tsien RY (1991). Fluorescence ratio imaging of cyclic-AMP in single cells. Nature 349, 694–697. DOI:10.1038/349694a0 1847505 10.1038/349694a0

[bibr2-25152564211008341] Allbritton NL Meyer T (1993). Localized calcium spikes and propagating calcium waves. Cell Calcium 14, 691–697. DOI:10.1016/0143-4160(93)90095-n 8131187 10.1016/0143-4160(93)90095-n

[bibr3-25152564211008341] Aston D Capel RA Ford KL Christian HC Mirams GR Rog-Zielinska EA Kohl P Galione A Burton RAB Terrar DA (2017). High resolution structural evidence suggests the sarcoplasmic reticulum forms microdomains with acidic stores (lysosomes) in the heart. Sci Rep 7, 40620. DOI:10.1038/srep40620 28094777 10.1038/srep40620 PMC5240626

[bibr4-25152564211008341] Averaimo S Assali A Ros O Couvet S Zagar Y Genescu I Rebsam A Nicol X (2016). A plasma membrane microdomain compartmentalizes ephrin-generated cAMP signals to prune developing retinal axon arbors. Nat Commun 7, 12896. DOI:10.1038/ncomms12896 27694812 10.1038/ncomms12896 PMC5059439

[bibr5-25152564211008341] Bare DJ Kettlun CS Liang M Bers DM Mignery GA (2005). Cardiac type 2 inositol 1,4,5-trisphosphate receptor: interaction and modulation by calcium/calmodulin-dependent protein kinase II. J Biol Chem 280, 15912–15920. DOI:10.1074/jbc.M41421220 15710625 10.1074/jbc.M414212200

[bibr6-25152564211008341] Bernabucci M Zhuo M (2016). Calcium activated adenylyl cyclase AC8 but not AC1 is required for prolonged behavioral anxiety. Mol Brain 9, 60. DOI:10.1186/s13041-016-0239-x 27234425 10.1186/s13041-016-0239-x PMC4884383

[bibr7-25152564211008341] Berridge MJ Bootman MD Roderick HL (2003). Calcium signaling: dynamics, homeostasis and remodelling. Nat Rev Mol Cell Biol 4, 517–529. DOI: 10.1038/nrm1155 12838335 10.1038/nrm1155

[bibr8-25152564211008341] Berridge MJ Irvine RF (1984). Inositol trisphosphate, a novel second messenger in cellular signal transduction. Nature 312, 315–321. DOI:10.1038/312315a0 6095092 10.1038/312315a0

[bibr9-25152564211008341] Bers DM Xiang YK Zaccolo M (2019). Whole-cell cAMP and PKA activity are epiphenomena, nanodomain signaling matters. Physiology 34, 240–249. DOI:10.1152/physiol.00002.2019 31165682 10.1152/physiol.00002.2019 PMC6863374

[bibr10-25152564211008341] Betzenhauser MJ Fike JL Wagner LE 2nd Yule DI (2009). Protein kinase A increases type-2 inositol 1,4,5-trisphosphate receptor activity by phosphorylation of serine 937. J Biol Chem 284, 25116–25125. DOI:10.1074/jbc.M109.010132 19608738 10.1074/jbc.M109.010132 PMC2757215

[bibr11-25152564211008341] Blatter LA (2017). The intricacies of atrial calcium cycling during excitation-contraction coupling. J Gen Physiol 149, 857–865. DOI:10.1085/jgp.201711809 28798277 10.1085/jgp.201711809 PMC5583713

[bibr12-25152564211008341] Blum AL Li W Cressy M Dubnau J (2009). Short- and long-term memory in *Drosophila* require cAMP signaling in distinct neuron types. Curr Biol 19, 1341–1350. DOI:10.1016/j.cub.2009.07.016 19646879 10.1016/j.cub.2009.07.016 PMC2752374

[bibr13-25152564211008341] Burgess GM Bird GS Obie JF Putney JW Jr (1991). The mechanism for synergism between phospholipase C- and adenylylcyclase-linked hormones in liver. Cyclic AMP-dependent kinase augments inositol trisphosphate-mediated calcium mobilization without increasing the cellular levels of inositol polyphosphates. J Biol Chem 266, 4772–4781. 1848225

[bibr14-25152564211008341] Burrell KM Molenaar P Dawson PJ Kaumann AJ (2000). Contractile and arrhythmic effects of endothelin receptor agonists in human heart in vitro: blockade with SB 209670. J Pharmacol Exp Ther 292, 449–459. 10604982

[bibr15-25152564211008341] Bychkov R Juhaszova M Tsutsui K Coletta C Stern MD Maltsev VA Lakatta EG (2020). Synchronized cardiac impulses emerge from heterogeneous local calcium signals within and among cells of pacemaker tissue. Jacc-Clin Electrophysiol 6, 907–931. DOI:10.1016/j.jacep.2020.06.022 32819526 10.1016/j.jacep.2020.06.022 PMC9665107

[bibr16-25152564211008341] Cao K Xia X Shan Q Chen Z Chen X Huang Y (2002). Changes of sarcoplamic reticular Ca(2+)-ATPase and IP(3)-I receptor mRNA expression in patients with atrial fibrillation. Chin Med J 115, 664–667. 12133531

[bibr17-25152564211008341] Capel RA Bolton EL Lin WK Aston D Wang Y Liu W Wang X Burton RA Bloor-Young D Shade KT Ruas M Parrington J Churchill GC Lei M Galione A Terrar DA (2015). Two-pore channels (TPC2s) and nicotinic acid adenine dinucleotide phosphate (NAADP) at lysosomal-sarcoplasmic reticular junctions contribute to acute and chronic β-adrenoceptor signaling in the heart. J Biol Chem 290, 30087–30098. DOI:10.1074/jbc.M115.684076 26438825 10.1074/jbc.M115.684076 PMC4705968

[bibr18-25152564211008341] Capel R A , Bose S J , Collins T P , Rajasundaram S , Ayagama T , Zaccolo M , Burton B , Terrar D A (2021). IP3-Mediated calcium Release Regulates Atrial calcium Transients and Pacemaker Function by Stimulation of Adenylyl Cyclases. Am J Physiol Heart Circ Physiol, 320, H95–H107. https://doi.org/10.1152/ajpheart.00380.2020 33064562 33064562 PMC7864251

[bibr19-25152564211008341] Capel RA Mu-u-Min R Aston D Ruas M Christian H Galione A Terrar DA Burton RAB (2020). The role of NAADP-mediated endo-lysosomal calcium release in the cardiac atria. Biophys J 118, 568A–568A.

[bibr20-25152564211008341] Capel RA Terrar DA (2015). The importance of Ca(2+)-dependent mechanisms for the initiation of the heartbeat. Front Physiol 6, 80. DOI:10.3389/fphys.2015.00080 25859219 10.3389/fphys.2015.00080 PMC4373508

[bibr21-25152564211008341] Chen WQ Wang RW Chen BY Zhong XW Kong HH Bai YL Zhou Q Xie CH Zhang JQ Guo A , et al. (2014). The ryanodine receptor store-sensing gate controls calcium waves and calcium-triggered arrhythmias. Nat Med 20, 184–192. DOI:10.1038/nm.3440 24441828 10.1038/nm.3440 PMC4269524

[bibr22-25152564211008341] Collins TP Bayliss R Churchill GC Galione A Terrar DA (2011). NAADP influences excitation-contraction coupling by releasing calcium from lysosomes in atrial myocytes. Cell Calcium 50, 449–458. DOI:0.1016/j.ceca.2011.07.007 21906808 10.1016/j.ceca.2011.07.007

[bibr23-25152564211008341] Collins TP Terrar DA (2012). Ca(2+)-stimulated adenylyl cyclases regulate the L-type Ca(2+) current in guinea-pig atrial myocytes. J Physiol 590, 1881–1893. DOI:110.1113/jphysiol.2011.227066 22351635 10.1113/jphysiol.2011.227066 PMC3573310

[bibr24-25152564211008341] Cooper DMF Mons N Karpen JW (1995). Adenylyl cyclases and the interaction between calcium and cAMP signaling. Nature 374, 421–424. DOI:10.1038/374421a0 7700350 10.1038/374421a0

[bibr25-25152564211008341] Cooper DMF Tabbasum VG (2014). Adenylate cyclase-centred microdomains. Biochem J 462, 199–213. DOI:10.1042/BJ20140560 25102028 10.1042/BJ20140560

[bibr26-25152564211008341] Dessauer CW (2009). Adenylyl cyclase-A-kinase anchoring protein complexes: the next dimension in cAMP signaling. Mol Pharmacol 76, 935–941. DOI:10.1124/mol.109.059345 19684092 10.1124/mol.109.059345 PMC2774998

[bibr27-25152564211008341] Dessauer CW Watts VJ Ostrom RS Conti M Dove S Seifert R (2017). International union of basic and clinical pharmacology. CI. Structures and small molecule modulators of mammalian adenylyl cyclases. Pharmacol Rev 69, 93–139. DOi:10.1124/pr.116.013078 28255005 10.1124/pr.116.013078 PMC5394921

[bibr28-25152564211008341] Domeier TL Zima AV Maxwell JT Huke S Mignery GA Blatter LA (2008). IP3 receptor-dependent calcium release modulates excitation-contraction coupling in rabbit ventricular myocytes. Am J Physiol Heart Circ Physiol 294, H596–H604. DOI:10.1152/ajpheart.01155.2007 18055509 10.1152/ajpheart.01155.2007

[bibr29-25152564211008341] Fabiato A (1992). Two kinds of calcium-induced release of calcium from the sarcoplasmic reticulum of skinned cardiac cells. Adv Exp Med Biol 311, 245–262. DOI:10.1007/978-1-4615-3362-7_18 1529757 10.1007/978-1-4615-3362-7_18

[bibr30-25152564211008341] Gassanov N Brandt MC Michels G Lindner M Er F Hoppe UC (2006). Angiotensin II-induced changes of calcium sparks and ionic currents in human atrial myocytes: potential role for early remodeling in atrial fibrillation. Cell Calcium 39, 175–186. DOI:10.1016/j.ceca.2005.10.008 16303176 10.1016/j.ceca.2005.10.008

[bibr31-25152564211008341] Guellich A Mehel H Fischmeister R (2014). Cyclic AMP synthesis and hydrolysis in the normal and failing heart. Pflugers Archiv Eur J Physiol 466, 1163–1175. DI:10.1007/s00424-014-1515-1 24756197 10.1007/s00424-014-1515-1

[bibr32-25152564211008341] Heijman J Voigt N Nattel S Dobrev D (2014). Cellular and molecular electrophysiology of atrial fibrillation initiation, maintenance, and progression. Circ Res 114, 1483–1499. DOI:10.1161/CIRCRESAHA.114.302226 24763466 10.1161/CIRCRESAHA.114.302226

[bibr33-25152564211008341] Hisatsune C Mikoshiba K (2017). IP(3) receptor mutations and brain diseases in human and rodents. J Neurochem 141, 790–807. DOI:10.1111/jnc.13991 28211945 10.1111/jnc.13991

[bibr34-25152564211008341] Hohendanner F Walther S Maxwell JT Kettlewell S Awad S Smith GL Lonchyna VA Blatter LA (2015). Inositol-1,4,5-trisphosphate induced calcium release and excitation-contraction coupling in atrial myocytes from normal and failing hearts. J Physiol 593, 1459–1477. DOI:10.1113/jphysiol.2014.283226 25416623 10.1113/jphysiol.2014.283226 PMC4376424

[bibr35-25152564211008341] Ju YK Liu J Lee BH Lai D Woodcock EA Lei M Cannell MB Allen DG (2011). Distribution and functional role of inositol 1,4,5-trisphosphate receptors in mouse sinoatrial node. Circ Res 109, 848–857. DOI:10.1161/CIRCRESAHA.111.243824 21852551 10.1161/CIRCRESAHA.111.243824

[bibr36-25152564211008341] Kang TM Hilgemann DW (2004). Multiple transport modes of the cardiac Na+/calcium exchanger. Nature 427, 544–548. DOI:10.1038/nature02271 14765196 10.1038/nature02271

[bibr37-25152564211008341] Kaplan P Jurkovicova D Babusikova E Hudecova S Racay P Sirova M Lehotsky J Drgova A Dobrota D Krizanova O (2007). Effect of aging on the expression of intracellular Ca(2+) transport proteins in a rat heart. Mol Cell Biochem 301, 219–226. DOI:10.1007/s11010-007-9414-9 17549608 10.1007/s11010-007-9414-9

[bibr38-25152564211008341] Kapoor N Tran A Kang J Zhang R Philipson KD Goldhaber JI (2015). Regulation of calcium clock-mediated pacemaking by inositol-1,4,5-trisphosphate receptors in mouse sinoatrial nodal cells. J Physiol 593, 2649–2663. DOI:10.1113/JP270082 25903031 10.1113/JP270082 PMC4500350

[bibr39-25152564211008341] Kapur N Banach K (2007). Inositol-1,4,5-trisphosphate-mediated spontaneous activity in mouse embryonic stem cell-derived cardiomyocytes. J Physiol 581, 1113–1127. DOI:10.1113/jphysiol.2006.125955 17379641 10.1113/jphysiol.2006.125955 PMC2170837

[bibr40-25152564211008341] Kentish JC Barsotti RJ Lea TJ Mulligan IP Patel JR Ferenczi MA (1990). Calcium release from cardiac sarcoplasmic reticulum induced by photorelease of calcium or Ins(1,4,5)P3. Am J Physiol 258, H610–H615. 2309921 10.1152/ajpheart.1990.258.2.H610

[bibr41-25152564211008341] Kockskämper J Zima AV Roderick HL Pieske B Blatter LA Bootman MD (2008). Emerging roles of inositol 1,4,5-trisphosphate signaling in cardiac myocytes. J Mol Cell Cardiol 45, 128–147. 18603259 10.1016/j.yjmcc.2008.05.014 PMC2654363

[bibr42-25152564211008341] Lakatta EG Maltsev VA Vinogradova TM (2010). A coupled SYSTEM of intracellular calcium clocks and surface membrane voltage clocks controls the timekeeping mechanism of the heart's pacemaker. Circ Res 106, 659–673. DOI:10.1161/CIRCRESAHA.109.206078 20203315 10.1161/CIRCRESAHA.109.206078 PMC2837285

[bibr43-25152564211008341] Lin WK Bolton EL Cortopassi WA Wang YW O'Brien F Maciejewska M Jacobson MP Garnham C Ruas M Parrington J , et al. (2017). Synthesis of the calcium - mobilizing messengers NAADP and cADPR by intracellular CD38 enzyme in the mouse heart: role in beta-adrenoceptor signaling. J Biol Chem 292, 13243–13257. DOI:10.1074/jbc.M117.789347 28539361 10.1074/jbc.M117.789347 PMC5555186

[bibr44-25152564211008341] Lipp P Laine M Tovey SC Burrell KM Berridge MJ Li W Bootman MD (2000). Functional InsP3 receptors that may modulate excitation-contraction coupling in the heart. Curr Biol 10, 939–942. DOI:10.1016/s0960-9822(00)00624-2 10959844 10.1016/s0960-9822(00)00624-2

[bibr45-25152564211008341] Mangoni ME Couette B Bourinet E Platzer J Reimer D Striessnig J Nargeot J (2003). Functional role of L-type Ca(v)13Ca(2+) channels in cardiac pacemaker activity. Proc Natl Acad Sci U S A 100, 5543–5548. 12700358 10.1073/pnas.0935295100 PMC154381

[bibr46-25152564211008341] Mangoni ME Couette B Marger L Bourinet E Striessnig J Nargeot J (2006). Voltage-dependent calcium channels and cardiac pacemaker activity: from ionic currents to genes. Progr Biophys Mol Biol 90, 38–63. DOI:10.1016/j.pbiomolbio.2005.05.003 10.1016/j.pbiomolbio.2005.05.003 15979127

[bibr47-25152564211008341] Marx SO Kurokawa J Reiken S Motoike H D'Armiento J Marks AR Kass RS (2002). Requirement of a macromolecular signaling complex for beta adrenergic receptor modulation of the KCNQ1-KCNE1 potassium channel. Science 295, 496–499. DOI:10.1126/science.1066843 11799244 10.1126/science.1066843

[bibr48-25152564211008341] Mattick P Parrington J Odia E Simpson A Collins T Terrar D (2007). calcium-stimulated adenylyl cyclase isoform AC1 is preferentially expressed in guinea-pig sino-atrial node cells and modulates the I(f) pacemaker current. J Physiol 582, 1195–1203. DOI:10.1113/jphysiol.2007.133439 17540702 10.1113/jphysiol.2007.133439 PMC2075242

[bibr49-25152564211008341] Molina CE Leroy J Richter W Xie M Scheitrum C Lee IO Maack C Rucker-Martin C Donzeau-Gouge P Verde I , et al. (2012). Cyclic adenosine monophosphate phosphodiesterase type 4 protects against atrial arrhythmias. J Am Coll Cardiol 59, 2182–2190. DOI:10.1016/j.jacc.2012.01.060 22676938 10.1016/j.jacc.2012.01.060

[bibr50-25152564211008341] Monterisi S Lobo MJ Livie C Castle JC Weinberger M Baillie G Surdo NC Musheshe N Stangherlin A Gottlieb E , et al. (2017). PDE2A2 regulates mitochondria morphology and apoptotic cell death via local modulation of cAMP/PKA signaling. eLife 6, e21374. DOI:10.7554/eLife.21374 28463107 10.7554/eLife.21374 PMC5423767

[bibr51-25152564211008341] Méry A Aimond F Ménard C Mikoshiba K Michalak M Pucéat M (2005). Initiation of embryonic cardiac pacemaker activity by inositol 1,4,5-trisphosphate-dependent calcium signaling. Mol Biol Cell 16, 2414–2423. DOI:10.1091/mbc.e04-10-0883 15758029 10.1091/mbc.E04-10-0883 PMC1087245

[bibr52-25152564211008341] Nattel S Burstein B Dobrev D (2008). Atrial remodeling and atrial fibrillation: mechanisms and implications. Circ Arrhythmia Electrophysiol 1, 62–73. DOI:10.1161/CIRCEP.107.754564 10.1161/CIRCEP.107.754564 19808395

[bibr53-25152564211008341] Nattel S Dobrev D (2012). The multidimensional role of calcium in atrial fibrillation pathophysiology: mechanistic insights and therapeutic opportunities. Eur Heart J 33, 1870–1877. DOI:10.1093/eurheartj/ehs079 22507975 10.1093/eurheartj/ehs079

[bibr54-25152564211008341] Nazarov IB Schofield CJ Terrar DA (2015). Contributions of cardiac “funny” (f) channels and sarcoplasmic reticulum calcium in regulating beating rate of mouse and guinea pig sinoatrial node. Physiol Rep 3, e12561. DOI:10.14814/phy2.12561 26660545 10.14814/phy2.12561 PMC4760437

[bibr55-25152564211008341] Nosek TM Williams MF Zeigler ST Godt RE (1986). Inositol trisphosphate enhances calcium release in skinned cardiac and skeletal muscle. Am J Physiol 250, C807–C811. DOI:10.1152/ajpcell.1986.250.5.C807 3085514 10.1152/ajpcell.1986.250.5.C807

[bibr56-25152564211008341] Pieske B Kockskämper J (2002). Alternans goes subcellular: a “disease” of the ryanodine receptor? Circ Res 91, 553–555. DOI:10.1161/01.res.0000036862.37203.f4 12364380 10.1161/01.res.0000036862.37203.f4

[bibr57-25152564211008341] Sassone-Corsi P (2012). The Cyclic AMP Pathway. Cold Spring Harbor Perspectives in Biology, 4, a011148–a011148. 10.1101/cshperspect.a011148 23209152 PMC3504441

[bibr58-25152564211008341] Scoote M Williams AJ (2004). Myocardial calcium signaling and arrhythmia pathogenesis. Biochem Biophys Res Commun 322, 1286–1309. 15336976 10.1016/j.bbrc.2004.08.034

[bibr59-25152564211008341] Streb H Irvine RF Berridge MJ Schulz I (1983). Release of calcium from a nonmitochondrial intracellular store in pancreatic acinar cells by inositol-1,4,5-trisphosphate. Nature 306, 67–69. 6605482 10.1038/306067a0

[bibr60-25152564211008341] Sunahara RK Taussig R (2002). Isoforms of mammalian adenylyl cyclase: multiplicities of signaling. Mol Intervent 2, 168–184. DOI:10.1124/mi.2.3.168 10.1124/mi.2.3.168 14993377

[bibr61-25152564211008341] Surdo NC Berrera M Koschinski A Brescia M Machado MR Carr C Wright P Gorelik J Morotti S Grandi E , et al. (2017). FRET biosensor uncovers cAMP nano-domains at beta-adrenergic targets that dictate precise tuning of cardiac contractility. Nat Commun 8, 15031. DOI:10.1038/ncomms15031 28425435 10.1038/ncomms15031 PMC5411486

[bibr62-25152564211008341] Taylor CW (2017). Regulation of IP(3) receptors by cyclic AMP. Cell Calcium 63, 48–52. DOI:10.1016/j.ceca.2016.10.005 27836216 10.1016/j.ceca.2016.10.005 PMC5471599

[bibr63-25152564211008341] Terrar DA (2020). Calcium signaling in the heart. Adv Exp Med Biol 1131, 395–443. DOI:10.1007/978-3-030-12457-1_16 31646519 10.1007/978-3-030-12457-1_16

[bibr64-25152564211008341] Terrin A Di Benedetto G Pertegato V Cheung YF Baillie G Lynch MJ Elvassore N Prinz A Herberg FW Houslay MD Zaccolo M (2006). PGE(1) stimulation of HEK293 cells generates multiple contiguous domains with different cAMP: role of compartmentalized phosphodiesterases. J Cell Biol 175, 441–451. DOI:10.1083/jcb.200605050 17088426 10.1083/jcb.200605050 PMC2064521

[bibr65-25152564211008341] Tovey SC Dedos SG Taylor EJ Church JE Taylor CW (2008). Selective coupling of type 6 adenylyl cyclase with type 2 IP3 receptors mediates direct sensitization of IP3 receptors by cAMP. J Cell Biol 183, 297–311. DOI:10.1083/jcb.200803172 18936250 10.1083/jcb.200803172 PMC2568025

[bibr66-25152564211008341] Toyoda F Mesirca P Dubel S Ding WG Striessnig J Mangoni ME Matsuura H (2017). Ca(V)1.3 L-type calcium channel contributes to the heartbeat by generating a dihydropyridine-sensitive persistent Na+ current. Sci Rep 7, 1:7869. DOI:10.1038/s41598-017-08191-8 28801600 10.1038/s41598-017-08191-8 PMC5554211

[bibr67-25152564211008341] Vadakkan KI Wang H Ko SW Zastepa E Petrovic MJ Sluka KA Zhuo M (2006). Genetic reduction of chronic muscle pain in mice lacking calcium/calmodulin-stimulated adenylyl cyclases. Mol Pain 2, 7. DOI:10.1186/1744-8069-2-7 16503978 10.1186/1744-8069-2-7 PMC1395303

[bibr68-25152564211008341] Van Wagoner DR Lindsay BD (2012). Phosphodiesterase-4 activity: a critical modulator of atrial contractility and arrhythmogenesis. J Am Coll Cardiol 59, 2191–2192. DOI:10.1016/j.jacc.2012.03.027 22676939 10.1016/j.jacc.2012.03.027 PMC3671593

[bibr69-25152564211008341] Villacres EC Wu Z Hua W Nielsen MD Watters JJ Yan C Beavo J Storm DR (1995). Developmentally expressed Ca(2+)-sensitive adenylyl cyclase activity is disrupted in the brains of type I adenylyl cyclase mutant mice. J Biol Chem 270, 14352–14357. DOI:10.1074/jbc.270.24.14352 7782295 10.1074/jbc.270.24.14352

[bibr70-25152564211008341] Vinogradova TM Lakatta EG (2011). Basal phospholipase C (PLC) activation is obligatory for cardiac pacemaker activity. Biophys J 100, 517a.21244848

[bibr71-25152564211008341] Vinogradova TM Lyashkov AE Zhu W Ruknudin AM Sirenko S Yang D Deo S Barlow M Johnson S Caffrey JL , et al. (2006). High basal protein kinase A-dependent phosphorylation drives rhythmic internal calcium store oscillations and spontaneous beating of cardiac pacemaker cells. Circ Res 98, 505–514. DOI:10.1161/01.RES.0000204575.94040.d1 16424365 10.1161/01.RES.0000204575.94040.d1

[bibr72-25152564211008341] Wachten S Masada N Ayling LJ Ciruela A Nikolaev VO Lohse MJ Cooper DMF (2010). Distinct pools of cAMP centre on different isoforms of adenylyl cyclase in pituitary-derived GH(3)B(6) cells. J Cell Sci 123, 95–106. DOI:10.1242/jcs.058594 20016070 10.1242/jcs.058594 PMC2794711

[bibr73-25152564211008341] Wang H Xu H Wu LJ Kim SS Chen T Koga K Descalzi G Gong B Vadakkan KI Zhang X , et al. (2011). Identification of an adenylyl cyclase inhibitor for treating neuropathic and inflammatory pain. Sci Transl Med 3, 65ra63. DOI:10.1126/scitranslmed.3001269 10.1126/scitranslmed.3001269 21228397

[bibr74-25152564211008341] Wang YG Dedkova EN Ji X Blatter LA Lipsius SL (2005). Phenylephrine acts via IP3-dependent intracellular NO release to stimulate L-type calcium current in cat atrial myocytes. J Physiol 567, 143–157. DOI:10.1113/jphysiol.2005.090035 15946966 10.1113/jphysiol.2005.090035 PMC1474159

[bibr75-25152564211008341] Wei F Qiu CS Kim SJ Muglia L Maas JW Pineda VV Xu HM Chen ZF Storm DR Muglia LJ Zhuo M (2002). Genetic elimination of behavioral sensitization in mice lacking calmodulin-stimulated adenylyl cyclases. Neuron 36, 713–726. DOI:10.1016/s0896-6273(02)01019-x 12441059 10.1016/s0896-6273(02)01019-x

[bibr76-25152564211008341] Wei J Zhao AZ Chan GCK Baker LP Impey S Beavo JA Storm DR (1998). Phosphorylation and inhibition of olfactory adenylyl cyclase by CaM kinase II in neurons: a mechanism for attenuation of olfactory signals. Neuron 21, 495–504. DOI:10.1016/s0896-6273(00)80561-9 9768837 10.1016/s0896-6273(00)80561-9

[bibr77-25152564211008341] Wiggins SV Steegborn C Levin LR Buck J (2018). Pharmacological modulation of the CO2/HCO3-/pH-, calcium-, and ATP-sensing soluble adenylyl cyclase. Pharmacol Ther 190, 173–186. DOI: 10.1016/j.pharmthera.2018.05.008 29807057 10.1016/j.pharmthera.2018.05.008 PMC6484840

[bibr78-25152564211008341] Wong ST Trinh K Hacker B Chan GCK Lowe G Gaggar A Xia ZG Gold GH Storm DR (2000). Disruption of the type III adenylyl cyclase gene leads to peripheral and behavioral anosmia in transgenic mice. Neuron 27, 487–497. DOI:10.1016/s0896-6273(00)00060-x 11055432 10.1016/s0896-6273(00)00060-x

[bibr79-25152564211008341] Xia Z Storm DR (1997). Calmodulin-regulated adenylyl cyclases and neuromodulation. Curr Opin Neurobiol 7, 391–396. DOI:10.1016/s0959-4388(97)80068-2 9232797 10.1016/s0959-4388(97)80068-2

[bibr80-25152564211008341] Yamda J Ohkusa T Nao T Ueyama T Yano M Kobayashi S Hamano K Esato K Matsuzaki M (2001). Up-regulation of inositol 1,4,5 trisphosphate receptor expression in atrial tissue in patients with chronic atrial fibrillation. J Am Coll Cardiol 37, 1111–1119. DOI:10.1016/s0735-1097(01)01144-5 11263617 10.1016/s0735-1097(01)01144-5

[bibr81-25152564211008341] Younes A Lyashkov AE Graham D Sheydina A Volkova MV Mitsak M Vinogradova TM Lukyanenko YO Li Y Ruknudin AM , et al. (2008). Ca(2+) -stimulated basal adenylyl cyclase activity localization in membrane lipid microdomains of cardiac sinoatrial nodal pacemaker cells. J Biol Chem 283, 14461–14468. DOI:10.1074/jbc.M707540200 18356168 10.1074/jbc.M707540200 PMC2386925

[bibr82-25152564211008341] Yu HJ Ma H Green RD (1993). Calcium-entry via L-type calcium channels acts as a negative regulator of adenylyl-cyclase activity and cyclic-AMP levels in cardiac myocytes. Mol Pharmacol 44, 689–693. 7694067

[bibr83-25152564211008341] Zaccolo M Pozzan T (2002). Discrete microdomains with high concentration of cAMP in stimulated rat neonatal cardiac myocytes. Science 295, 1711–1715. DOI:10.1152/ajpheart.00630.2014 11872839 10.1126/science.1069982

[bibr84-25152564211008341] Zaccolo M Zerio A Lobo MJ (2021). Subcellular organization of the cAMP signaling pathway. Pharmacol Rev 73, 278–309. 33334857 10.1124/pharmrev.120.000086 PMC7770493

[bibr85-25152564211008341] Zhao Z Babu GJ Wen H Fefelova N Gordan R Sui X Yan L Vatner DE Vatner SF Xie LH (2015). Overexpression of adenylyl cyclase type 5 (AC5) confers a proarrhythmic substrate to the heart. Am J Physiol Heart Circ Physiol 308, H240–H249. DOI:10.1152/ajpheart.00630.2014 25485900 10.1152/ajpheart.00630.2014 PMC4312946

[bibr86-25152564211008341] Zhao ZH Zhang HC Xu Y Zhang P Li XB Liu YS Guo JH (2007). Inositol-1,4,5-trisphosphate and ryanodine-dependent calcium signaling in a chronic dog model of atrial fibrillation. Cardiology 107, 269–276. DOI:10.1159/000095517 16954684 10.1159/000095517

[bibr87-25152564211008341] Zima AV Blatter LA (2004). Inositol-1,4,5-trisphosphate-dependent Ca(2+) in cat atrial excitation-contraction coupling and arrhythmias. J Physiol 555, 607–615. DOI:10.1016/s0735-1097(01)01144-5 14754996 10.1113/jphysiol.2003.058529 PMC1664857

